# Oncogenic Metabolism Acts as a Prerequisite Step for Induction of Cancer Metastasis and Cancer Stem Cell Phenotype

**DOI:** 10.1155/2018/1027453

**Published:** 2018-12-23

**Authors:** Su Yeon Lee, Min Kyung Ju, Hyun Min Jeon, Yig Ji Lee, Cho Hee Kim, Hye Gyeong Park, Song Iy Han, Ho Sung Kang

**Affiliations:** ^1^Department of Molecular Biology, College of Natural Sciences, Pusan National University, Pusan 609-735, Republic of Korea; ^2^Department of Internal Medicine and Institute of Health Science, Gyeongsang National University School of Medicine and Hospital, Jinju 52828, Republic of Korea; ^3^DNA Identification Center, National Forensic Service, Seoul 158-707, Republic of Korea; ^4^Nanobiotechnology Center, Pusan National University, Pusan 609-735, Republic of Korea; ^5^The Division of Natural Medical Sciences, College of Health Science, Chosun University, Gwangju 501-759, Republic of Korea

## Abstract

Metastasis is a major obstacle to the efficient and successful treatment of cancer. Initiation of metastasis requires epithelial-mesenchymal transition (EMT) that is regulated by several transcription factors, including Snail and ZEB1/2. EMT is closely linked to the acquisition of cancer stem cell (CSC) properties and chemoresistance, which contribute to tumor malignancy. Tumor suppressor p53 inhibits EMT and metastasis by negatively regulating several EMT-inducing transcription factors and regulatory molecules; thus, its inhibition is crucial in EMT, invasion, metastasis, and stemness. Metabolic alterations are another hallmark of cancer. Most cancer cells are more dependent on glycolysis than on mitochondrial oxidative phosphorylation for their energy production, even in the presence of oxygen. Cancer cells enhance other oncogenic metabolic pathways, such as glutamine metabolism, pentose phosphate pathway, and the synthesis of fatty acids and cholesterol. Metabolic reprogramming in cancer is regulated by the activation of oncogenes or loss of tumor suppressors that contribute to tumor progression. Oncogenic metabolism has been recently linked closely with the induction of EMT or CSC phenotypes by the induction of several metabolic enzyme genes. In addition, several transcription factors and molecules involved in EMT or CSCs, including Snail, Dlx-2, HIF-1*α*, STAT3, TGF-*β*, Wnt, and Akt, regulate oncogenic metabolism. Moreover, p53 induces metabolic change by directly regulating several metabolic enzymes. The collective data indicate the importance of oncogenic metabolism in the regulation of EMT, cell invasion and metastasis, and adoption of the CSC phenotype, which all contribute to malignant transformation and tumor development. In this review, we highlight the oncogenic metabolism as a key regulator of EMT and CSC, which is related with tumor progression involving metastasis and chemoresistance. Targeting oncometabolism might be a promising strategy for the development of effective anticancer therapy.

## 1. Introduction

Cancer cells can acquire multiple biological capabilities to overcome their multistage resistance during carcinogenesis. Hanahan and Weinberg defined ten hallmarks of cancer that alter cell physiology to enhance malignant transformation: (1) sustained proliferation, (2) evasion of growth suppression, (3) cell death resistance, (4) replicative immortality, (5) evasion of immune destruction, (6) tumor-promoting inflammation, (7) activation of invasion and metastasis, (8) induction of angiogenesis, (9) genome instability, and (10) alteration of metabolism [1, 2].

One of the most important differences between benign and malignant tumors is metastatic potential [1–3]. A malignant tumor can spread to surrounding tissue or more distantly in the body by invasion and metastasis, whereas benign tumors cannot spread and tend to remain localized. Among several cancer hallmarks, metastasis is a major cause (90%) of deaths from malignant tumors. Thus, metastasis could be a major target for efficient and successful treatment of cancer [3, 4]. However, the regulatory mechanisms underlying metastasis in tumors need to be understood before the efficacy of cancer therapy targeting metastasis is improved.

Metastasis is regulated by intrinsic and extrinsic cancer cell mechanisms. Extrinsic mechanisms, such as the metabolic, stromal, and immunological microenvironments, can control metastasis [4–8]. According to the “seed and soil” theory, cancer cells display the propensity to find a microenvironment with characteristics that favor for their colonization and subsequent metastasis, since it is difficult for cancer cells to survive outside their region of origin [9, 10]. Seed and soil factors, such as extracellular vesicles and exosomes, play a critical role in remodeling the primary microenvironment and in priming the secondary microenvironment, thereby inducing the formation of a pre-metastatic niche [10]. Extracellular vesicles and exosomes contain cargo molecules that include nucleic acids, proteins, lipids, messenger RNA (mRNA), microRNAs (miRNAs), and noncoding RNAs (ncRNAs), which provide intercellular communication between neighboring and distant cells both locally and systemically. Cancer cells release these molecules to alter the host microenvironment to a pre-metastatic microenvironment [10–15]. Extrinsic mechanisms of cancer cells in metastasis have been reviewed elsewhere [4–15] and are not discussed in detail here.

A typical intrinsic mechanism of cancer cells is the epithelial-mesenchymal transition (EMT). The EMT plays an essential role in the initial step of metastasis. Many transcription factors, including Snail, Slug, zinc finger E-box-binding homeobox (ZEB)1/2, Twist-related protein (Twist) 1/2, E12/E47, hypoxia-inducible factor-1-alpha (HIF-1*α*), and distal-less homeobox 2 (Dlx-2), and several regulatory molecules contribute to EMT, invasion, and metastasis. Epithelial cells undergoing EMT exhibit phenotypic changes, including the loss of epithelial cell polarity and the gain of mesenchymal proteins [3, 16–23]. EMT confers increased metastatic ability and contributes to the acquisition of cancer stem cell (CSC) phenotypes [3, 19–23]. Besides their pro-metastatic roles, EMT-inducing transcription factors are involved in oncogenic transformation by regulating cancer cell stemness, protecting cancer cells from safeguard programs (senescence and apoptosis), determining resistance to chemotherapy, and promoting tumor angiogenesis and metabolic alterations [24, 25]. Recently, oncogenic metabolism has been shown to play important roles in the regulation of EMT, cell invasion and metastasis, and CSC phenotype, via induction of several metabolic enzyme genes.

In this review, we discuss oncogenic metabolism as a key regulator of EMT and CSC, which is related with metastasis and chemoresistance, and which in turn influences the malignancy of cancer cells.

## 2. EMT

### 2.1. EMT and Metastasis

EMT involves the loss of epithelial homeostasis with the acquisition of migratory mesenchymal capabilities. The characteristics of EMT include the downregulation of epithelial markers, such as E-cadherin, desmoplakin, mucin-1, cytokeratin-18, occludins, claudins, and zonula occludens-1 (ZO-1), and the acquisition of mesenchymal markers, such as N-cadherin, vimentin, fibronectin, vitronectin, *α*-smooth muscle actin (*α*-SMA), and fibroblast-specific protein 1. The foregoing molecules are usually used as EMT markers [3, 16–23].

Both EMT and the reverse process, mesenchymal-epithelial transition (MET), occur in the development of embryonic cells and cancerous cells. MET involves profound phenotypic changes, including the loss of migratory freedom with the expression of junction complexes. MET is essential for clonal outgrowth at metastatic sites. EMT and MET both are crucial in the invasive and metastatic properties of cancer cells [3, 17, 20–22]. EMT confers the capability of cancer cells to initiate tumor dissemination and metastasis through the acquisition of invasive and migratory capabilities [3, 16–23].

Transcription factors capable of inducing EMT play a crucial role in cancer progression, including tumor growth and drug resistance in cancer cells, as well as in promoting invasion and metastasis [3, 20, 22, 23].

### 2.2. EMT-Inducing Transcription Factors

EMT-inducing transcription factors include Snail/Slug, ZEB1/*δ*EF1, ZEB2/SIP1, Twist1/2, and E12/E47, which regulate the expression of proteins involved in cell polarity, cell-cell contacts, cytoskeletal structural maintenance, and extracellular matrix (ECM) degradation. A hallmark of EMT is a loss of E-cadherin in various cancers. These EMT-inducing transcription factors repress E-cadherin transcription by binding to the E-box elements of the E-cadherin gene promoter. Especially, Snail is involved in the initial cell-migratory phenotype and is regarded as an early marker of EMT [3, 17, 20–22, 24–27]. In normal cells, E-cadherin forms a complex with *β*-catenin, which plays a significant role in adherens junctions. The E-cadherin/*β*-catenin complex contributes to the maintenance of epithelial integrity. Loss of E-cadherin promotes *β*-catenin release and subsequent activation. When *β*-catenin translocates to the nucleus, it can directly transcriptionally activate EMT-associated target genes, including Slug, thereby facilitating EMT [28].

ZEB1 and 2 repress the expression of several cellular junction proteins, such as claudins and ZO-1, thereby promoting invasion and metastasis. Twist directly and indirectly affects other EMT-linked factors and then induces EMT. For example, Twist1 binds to the E-cadherin promoter to repress its expression but also induces Snail to inhibit E-cadherin [3, 24–27, 29].

The expressions of Snail and ZEB1/ZEB2/Twist are mutually dependent, although they can occur in different directions depending on the cell system. In many cases, Snail increases the levels of ZEB1 and ZEB2 proteins through both transcriptional and posttranscriptional mechanisms. In addition, Snail can regulate Twist1 protein and mRNA. Snail may be required for the initiation of EMT with subsequent consolidation by Twist and ZEB factors [29–32].

Several other transcription factors, including HIF-1*α* and Dlx-2, contribute to EMT. HIF-1*α* is a transcription factor that responds to low oxygen concentrations (hypoxia). HIF-1*α* has been strongly correlated with cell survival, proliferation, motility, EMT, metastasis, metabolism, pH regulation, ECM function, inflammatory cell recruitment, angiogenesis, chemotherapeutic resistance, and poor prognosis by regulating the expression of its target genes in several types of tumors [33–36]. HIF-1*α* suppresses E-cadherin expression by activating Snail, which promotes EMT [34]. HIF-1*α* also binds to *β*-catenin by competing with transcription factor 4 (TCF4), thereby inducing EMT in colorectal cancer [35]. In addition, HIF-1*α* promotes EMT and cancer metastasis by binding to the promoter of ZEB1 in colorectal cancer [36].

Dlx-2 is a homeobox transcription factor that is crucial for embryonic development, morphogenesis, and tissue homeostasis [37, 38]. Recently, Dlx-2 has been shown to play an important role in transforming growth factor-beta- (TGF-*β*-) and Wnt-induced EMT by Snail activation [22, 39, 40], indicating a crucial role for Dlx-2 in EMT, migration, and invasion. Dlx-2 levels are enhanced in many tumors, and it confers a poor prognosis [22, 39–52]. High expression levels of Dlx-2 positively affect tumor size, depth of invasion, and metastasis stages in several cancers, including gastric adenocarcinoma and hepatocellular carcinoma [42, 47, 48, 50, 52]. In addition, Dlx-2 is involved in shifting from the TGF-*β* tumor suppressive activity in early stages to tumor promoting activity in later stages [42]. Dlx1/2 genes also promote cell migration by repression of the expression of p21-activated kinase (PAK) 3, which is a key effector for adhesion turnover and protrusion dynamics [51]. In addition, Dlx-2 confers radioresistance and drug resistance [22, 41, 48]. In response to ionizing radiation, the expression of Dlx-2 is induced by activation of Smad2/3 and Dlx-2 contributes to the radiation-induced EMT and radioresistance in A549 and MDA-MB-231 cell lines [41]. Dlx-2 is increased by ionizing radiation-induced reactive oxygen species and is important in radiation-induced EMT by Snail activation [22].

Recently, it was also reported that Dlx-2 negatively regulates the growth, migration, and invasion of cells. Dlx-2 is regulated by p53-R273H, which exhibits a gain of function that promotes cell mobility and tumor metastasis. p53-R273H induces the downregulation of Dlx-2 and the upregulation of neuropilin 2 (NRP2) [53, 54], which act as a multifunctional coreceptor associated with tumor initiation, growth, and metastasis [55, 56]. The reduction of Dlx-2 promotes p53-R273H-induced cell growth, migration, and invasion and also induces the expression of NRP2. In addition, p53-R273H-induced tumor metastasis is prevented by knockdown of NRP2 in vivo. p53-R273H contributes to cell mobility, invasion, and tumor metastasis by increasing NRP expression through the repression of Dlx-2 [53, 54]. The collective data indicate that Dlx-2 has both pro-metastatic and antimetastatic activities depending on the cellular context.

### 2.3. EMT-Inducing Signal Pathways

EMT is controlled by a network of growth factors that include TGF-*β*, Wnt, epidermal growth factor (EGF), Notch, and Hedgehog and their associated signaling proteins, which include nuclear-factor kappa B (NF-*κ*B), extracellular signal-regulated kinase (ERK), and phosphatidylinositol 3-kinase (PI3K)/Akt. These signaling pathways are linked to tumorigenesis and tumor progression in response to stresses that include hypoxia, oncogenic or metabolic stress, inflammation, and physical constraints. These signals activate EMT-inducing transcription factors, including Snail, Slug, ZEB1/2, and Twist1/2 [18, 21, 22, 57–60].

TGF-*β* signaling activates Smad2 and 3, which form a complex with Smad4 and translocate to the nucleus. The complex induces target genes by the transcription of EMT-inducing transcription factors [57, 58, 60]. TGF-*β* signaling also induces the activation of GTPases, PI3K, and mitogen-activated protein kinase (MAPK) pathways in the Smad-independent pathway, thereby inducing EMT [61].

Wnt/*β*-catenin and Notch signaling pathways are important in the induction of EMT. These signaling pathways have been implicated in the activation of EMT-inducing transcription factors [18, 21, 57–59]. In addition, several other regulatory molecules, including leptin, interleukin- (IL-) 6, and IL-17, also activate EMT-inducing signaling pathways and then contribute to EMT [62–66]. Thus, numerous EMT-related signaling pathways contribute to the malignant transformation and aspects of tumor development that include invasion, metastasis, and CSC phenotype.

EMT programs are aberrantly activated in triple-negative breast cancer (TNBC) [67–70]. Breast cancer is a heterogeneous disease, which includes luminal A, luminal B, HER2-overexpressing, and TNBC (or basal-like) cancer, with pronounced cell plasticity. TNBC is the most lethal breast cancer subtype. It is characterized by the lack of estrogen receptor and progesterone receptor expression and human epidermal growth factor receptor amplification. These characteristics are caused by the contribution of a diverse range of factors that influence the induction of EMT in cancer cells through the process of dedifferentiation. In this process, cancer cells can acquire stem-like features and the capacity to migrate and invade [69]. Recently, the high expression of the c-Met growth factor receptor was described in TNBC. c-Met signaling pathways have also been implicated in the initiation of EMT. These observations suggest that the interplay between c-Met and EMT may be important in TNBC metastasis [70].

### 2.4. p53 and EMT/Metastasis

The tumor suppressor p53 is activated by various cellular stresses, including DNA damage, ribosomal stress, hypoxia, nutrient deprivation, and oncogene activation. Activated p53 exerts the tumor suppressive function through transcriptional regulation of target genes that regulate numerous cellular processes, such as cell cycle arrest, DNA repair, senescence, apoptosis, autophagy, antioxidant defense, mRNA translation, and feedback mechanisms [71–78].

p53 is one of the most potential regulators of metastasis. p53 binds to the promoters of a variety of genes associated with cell motility, adhesion, and invasion, thereby regulating the transcription of genes involved in metastasis [79–87]. p53 inhibits EMT and metastasis by negatively regulating several EMT-inducing transcription factors, including Snail, Slug, and Twist [80–82]. Furthermore, p53 represses Snail expression by inducing the expression of miR-34, thereby suppressing tumor cell migration and invasion [80, 81]. In addition, wild-type (wt) p53 suppresses cancer invasion and metastasis by inducing mouse double minute- (Mdm-) mediated Slug degradation [80, 82].

The functions of p53 are negatively regulated by Twist. Twist inhibits alternative reading frame tumor suppressor protein and then induces Mdm2-mediated p53 ubiquitination and degradation. Thus, Twist indirectly antagonizes p53 function to promote EMT [80, 88].

p53 acts as an antagonist of HIFs, which contribute to resistance to therapy and metastasis and are associated with poor survival rates in cancer patients. The activation of HIF-1-dependent signaling is suppressed by p53. The interplay between the p53 family and HIFs is important in cancer progression via the regulation of angiogenesis, the tumor microenvironment, dormancy, metastasis, and recurrence [79].

In addition, p53 regulates other molecules, including p21, Bcl-2 family proteins, KAI, and *β*-catenin, to inhibit EMT, invasion, and metastasis [83–87]. p53 is acts cooperatively with p21 to form a p21–p53–Slug–Mdm2 complex, which inhibits cell invasion [83]. Furthermore, the p53/p21 complex interacts with Bcl-2 family proteins and releases Bax from Bcl-XL, thereby increasing apoptosis and inhibiting invasion [84]. Cytoplasmic p53 also interacts with the Bcl-2 family of proteins and then inhibits complex I activity and reactive oxygen species (ROS) production, thereby inhibiting cell invasion [85]. p53 activates KAI, a tumor metastasis suppressor gene, which suppresses the metastatic process. The loss of the functional capabilities of p53 contributes to the progression of metastasis by the downregulation of the KAI1 gene [86]. p53 also prevents EMT and metastasis of hepatocellular carcinoma (HCC) cells by negatively regulating *β*-catenin [87].

In addition, tumor protein p53-inducible nuclear protein 1 (TP53INP1), also termed stress-induced protein, inhibits malignant tumor metastasis. In response to oxidative stress, TP53INP1 is a key regulator of p53. High expression of TP53INP1 negatively correlates with VE-cadherin, HIF-1*α*, and Snail expression. Under hypoxic conditions, TP53INP1 suppresses EMT and vasculogenic mimicry, which is the formation of a new tumor vascular supply system, by regulating the ROS/GSK-3*β*/Snail pathway in breast cancer, which is crucial role in cancer progression and metastasis [89].

The foregoing findings indicate that loss of p53 function is important in the progression of carcinogenesis. Indeed, p53 is the most frequently lost or mutated molecule in human cancer. More than half of human cancers display loss of p53 function through DNA mutations and different mechanisms. In human cancer, the majority of p53 mutations are missense mutations and mutant p53 protein accumulates to very high levels. p53 mutants are also associated with poor prognosis in many human tumors [71–75, 80, 90–92]. The loss of p53 or the expression of p53 gain-of-function mutants can promote tumor initiation and progression and also affect the metastatic potential of tumor cells by disrupting pathways, such as those of the Rho family of small GTPases [80].

p53 mediates tumorigenesis by regulating the expression of ncRNAs, such as miRNAs and long ncRNAs (lncRNAs). The wt p53 regulates the transcriptional expression and the biogenesis of specific miRNAs involved in cell cycle arrest, senescence, and apoptosis, as well as in the inhibition of metastasis, angiogenesis, and glycolysis [71, 93–95].

However, many mutant p53 have gain-of-function activities that are involved in regulation of the expression and the biogenesis of different miRNAs independent of wt p53, thereby promoting tumorigenesis [71, 94, 95].

A number of miRNAs target EMT-inducing transcription factors or EMT-activating pathways that control epithelial cell plasticity. For example, Snail and Slug are repressed by p53-dependent miR-34a/b/c. ZEB1 and ZEB2 are suppressed by the miR-200 family, including miR-200a/b/c, miR-141, and miR-429, thereby inhibiting EMT. Twist1 is negatively regulated by miR-186, which inhibits EMT [3, 17, 20, 21, 24, 29, 96].

Some miRNAs and EMT-inducing transcription factors form the most relevant networks that contribute to maintenance of the epithelial or mesenchymal state. Recent studies demonstrated that p53 regulates EMT-inducing transcription factors and miRNA loops, and the epigenetic regulation of miRNAs, to maintain the epithelial phenotype [29, 96].

p53 regulates the transcriptional expression of miR-34a, a key regulator of tumor suppression. p53-dependent regulation of miR-34a expression has been implicated in context-dependent feedback loops [71, 81, 97–99]. p53 prevents EMT by inhibiting Snail [81, 97], zinc finger protein 281 [98], and IL-6 receptor [99] via miR-34a. Aside from p53-driven miR-34a expression, miR-34a levels can be regulated in a p53-independent manner. Mechanisms responsible for p53-independent regulation can operate simultaneously with p53-dependent control [100, 101].

p53 regulates the transcriptional level of long intergenic ncRNA- (lincRNA-) p21. lincRNA-p21 represses the expression of p53 target genes [102]. In addition, p53 prevents EMT by inducing the anti-EMT lncRNAs, including tumor suppressor candidate 7, growth arrest-specific 5, and lincRNA-p21 [103–105]. These results suggest that p53 may contribute to EMT and metastasis by the regulation of miRNA and lncRNA. Therefore, p53-dependent regulation of miRNA and lncRNA may contribute to EMT and metastasis.

Several miRNAs, including miR-125b and miR-655, inhibit EMT in TNBC [106, 107]. miR-125b contributes to the activation of the c-Jun NH_2_-terminal kinase group of MAPKs and inhibits EMT of TNBC cells [106]. miR-655 expression is involved in lymph node metastasis in breast cancer. miR-655 overexpression upregulates cytokeratin and downregulates vimentin expression, inhibiting EMT. Furthermore, miR-655 negatively regulates Prrx1, a newly identified EMT inducer, and then inhibits the acquisition of the EMT phenotype in TNBC, thereby suppressing migration and invasion during cancer progression [107].

### 2.5. EMT and CSC/Chemoresistance

EMT has been closely linked to CSCs [23, 108–120]. The EMT mechanism helps the cells become metastatic and promotes the CSCs present in the heterogeneous tumor mass. Thus, EMT has emerged as a central driver of tumor malignancy [108–114]. Cancer cells undergoing EMT exhibit gene expression signatures and marker expression similar to CSC. The cells have more drug efflux pumps and antiapoptotic effects, indicating that activation of the EMT mechanism is involved in generating CSCs [23, 116]. Snail, ZEB, and Twist have been shown to acquire stemness properties [25, 27].

CSCs comprise a small subpopulation of cancer cells. These cells have stem cell-like properties; they are able to self-renew, generate differentiated daughter cells, and give rise to heterogeneous tumor tissue. CSCs have been found in several solid tumors, including breast, brain, colon, ovary, pancreas, prostate, and melanoma [121–127]. CSCs are associated with the initiation, progression, and recurrence of cancers. Many tumors harbor CSCs in dedicated niches, and their identification and eradication remain complicated [128–133]. Conventional chemotherapies kill differentiated or differentiating cancer cells, but CSC-like normal stem cells are more resistant to the therapy. Persistence of CSCs leads to tumor relapse and metastasis. The observations suggest that CSCs may be a potential therapeutic target to improve the survival and quality of life of cancer patients, especially those with metastatic disease [134–139].

CSCs are detected in solid and hematological tumors using markers that are specific for normal stem cells. The markers include CD133 (also known as PROM1), CD44, CD24, epithelial cell adhesion molecule (also known as epithelial specific antigen), THY1, ATP-binding cassette B5, and CD200 [140]. Given the potential importance of CSCs in cancer therapy, understanding the cellular mechanisms underlying the transformation of normal stem cells to CSCs is necessary to design drugs that target CSCs [131–133]. Eradicating CSCs is especially important since they can metastasize.

TGF-*β*1 induces stemness properties by increasing EMT markers (Slug, Twist1, *β*-catenin, and N-cadherin) and CSC markers (Oct4, Sox2, Nanog, and Klf4) in breast and lung cancer cells [117, 118]. The Notch and Wnt/*β*-catenin signaling pathways also promote the stemness characteristics of liver CSCs. Expression of transcription factors involving EMT (such as Snail) and stemness (such as Sox2 and Nanog) can be decreased by blocking the function of the Wnt/*β*-catenin and/or Notch [141]. This indicates that EMT-inducing signaling pathways play important roles in the acquisition of CSC phenotypes.

In addition, EMT-inducing transcription factors are able to mitigate p53-dependent tumor-suppressive functions and gain of stemness-related properties, creating a protumorigenic environment [24, 25]. A p53 gain of function has been associated with stemness [142]. p53 has important roles in pluripotent stem cells and pluripotent stem cell-like CSCs. p53 is also associated with the differentiation of mouse embryonic stem cells [143, 144].

p53 regulates several cell surface markers, including CD44 and CD133, which are associated with CSCs. Furthermore, p53 negatively regulates CD44 expression to regulate stemness. CD44 is involved in anchorage-independent growth and metastasis. p53 may contribute to growth inhibition and tumor suppression functions by directly repressing CD44 expression. Constitutive CD44 expression inhibits p53-dependent apoptosis and enhances genotoxic doxorubicin-resistant cell populations [80, 142, 145, 146]. In addition, transcriptional repression of CD133 by p53 contributes to the suppression of CSCs. CD133 has crucial roles in tumor cell proliferation, colony formation, and the expression of stemness genes, including NANOG, OCT4, SOX2, and c-MYC [142, 146]. Thus, p53 is important in the repopulating activity of tissue-specific stem cells [80, 147].

p53, focal adhesion kinase (FAK), and Nanog, a key embryonic stem cell (ESC) transcription factor, are interconnected in the regulation of CSCs. Nanog and FAK survival signaling pathways contribute to maintenance of CSCs. p53 upregulation blocks the Nanog and FAK survival signaling pathways, and p53 suppresses FAK and Nanog. FAK induces p53 degradation by binding Mdm2. Nanog maintains the CSC pool and blocks differentiation, cell cycle arrest, apoptosis, and cell growth by suppressing p53, thereby enhancing tumor growth. Nanog also leads to the upregulation of FAK, which in turn leads to the phosphorylation of Nanog. This cross-linked signaling plays an important role in cell motility and invasion of CSCs and contributes to tumor metastasis [148, 149].

In response to DNA damage, activated p53 also induces ESC differentiation by suppressing Nanog [143, 144]. In glial stem cells and CSCs, loss of p53 leads to induction of Hedgehog signaling and subsequent increase in Nanog production. Perturbed Hedgehog signaling regulates self-renewal associated with glioma stem cell fate and the transforming cell properties of CSCs by altering the fate of Nanog [148].

EMT is also involved in the generation of circulating tumor cells (CTCs). CTCs are cells left from the primary tumor. They are present in the bloodstream and can spread from the original tumor to distant locations. EMT and CSC phenotypes have been demonstrated in CTCs of some cancer patients. CTC subpopulations exhibit several phenotypes, such as epithelial, mesenchymal, and stem-like cell traits. Among these subpopulatons, CTCs with a mesenchymal phenotype presumably underwent EMT. In addition, CTCs with stem cell-like characteristics may be important drivers in tumor progression [120]. CTCs with EMT or stem cell features may be an indicator of cell populations that will be resistant to therapy.

In addition, EMT and CSC phenotypes contribute to chemoresistance in cancer cells. Chemoresistance increases the likelihood of cancer recurrence and metastasis. Intriguingly, chemotherapy can induce the EMT and CSC phenotypes. For example, cisplatin-resistant lung cancer cells exhibit enhanced EMT and CSC properties by the Akt/*β*-catenin/Snail signaling pathway [150, 151]. A p53 gain-of-function mutant that induces drug resistance was described [142]. Thus, the connection between the EMT program and acquisition of stem cell traits appears to be important in metastasis and chemoresistance in cancer cells and warrants further study.

### 2.6. EMT and Epithelial-Mesenchymal Plasticity

Cancer cells exhibit phenotypic plasticity, in which the cells switch back and forth among multiple phenotypes, including epithelial, mesenchymal, and hybrid epithelial/mesenchymal (E/M) phenotype(s), in response to signals. Phenotypic plasticity is associated with metabolism, immune evasion, invasion, and metastasis, thereby accelerating tumor progression [152–154].

E/M plasticity is a canonical example of phenotypic plasticity, which contributes to metastasis and drug resistance. Interestingly, cancer cells rarely display all or none of these transitions. Rather, partial EMT, or full EMT, and its reverse, MET, are evident. A hybrid E/M phenotype can often be observed in cancer cells. This phenotype combines various epithelial and mesenchymal morphological and/or molecular features. These hydrid cells can be markedly more tumorigenic and drug-resistant as compared to cells in a strongly full epithelial or mesenchymal state [152–156]. E/M plasticity may be controlled by various EMT core programs, which serve as a major mechanism for generating CSCs [157].

Highly metastatic TNBC tumors often contain cells with E/M plasticity, which lead to the reversible expression of epithelial or mesenchymal protein. E/M plasticity can induce mesenchymal CSCs that are very prone to migrate. The primary tumors in TNBC patients are effectively removed by standard care chemotherapy to eliminate the more proliferative epithelial cells, which are regarded as epithelial/non-CSCs. Epithelial/non-CSCs often lack the aggressive CSC properties. On the other hand, mesenchymal CSCs can grow slowly. Thus, chemotherapy can fail for mesenchymal CSCs [108, 158–160].

### 2.7. EMT and Cell Senescence

Cancer cells that undergo extensive proliferation can senesce through the loss of telomeres. Cellular senescence irreversibly arrests cell proliferation and is also induced by diverse stimuli, including oxidative stress, DNA-damaging agents, and activation of oncogenes, independent of telomere length [161–170]. The telomere shortening induced during replicative senescence elicits DNA damage [161]. Oncogene-induced senescence is also associated with DNA replication stress, leading to impaired replication forks and DNA damage [171–173].

DNA damage activates the DNA damage response (DDR) signaling. The key components of DDR are p53, ATM, ATR, and Chk1/2. DNA damage can occur in several different forms, including large chromosomal lesions, such as double-strand breaks (DSBs), and small, local lesions such as single-strand breaks (SSBs) [167, 168, 174]. DSBs are the most harmful form of DNA damage. After DSB generation, the DNA damage sensor MRN complex leads to the activation of *γ*H2AX by recruiting the protein kinase ATM. *γ*H2AX binds to mediator of DNA damage checkpoint 1 (MDC1) and p53 binding protein 1 (53BP1), inducing the activation of Chk2. In case of SSB, the 9-1-1 complex and TOPBP1 induce the activation of the protein kinase ATR, leading to the activation of Chk1. Subsequently, p53 is activated by ATM-Chk2 and ATR-Chk1 pathways [167–170, 173, 174].

Activated p53 regulates the transcription of several target genes. p53 transactivates many proapoptotic proteins including the BCL2 family (BAX, BID, PUMA, and NOXA) to induce apoptosis [72, 175, 176]. In addition, as mentioned above, p53 induces the expression of p21, which activates retinoblastoma protein (RB) through inhibition of cyclin-dependent kinase (CDK) 2. p53, p21, and RB cause transient cell cycle arrest and senescence by interacting with the activities of other molecules [170, 177, 178]. Transient cell cycle arrest, apoptosis, and senescence suppress tumorigenesis [168, 177].

**Figure 1:**
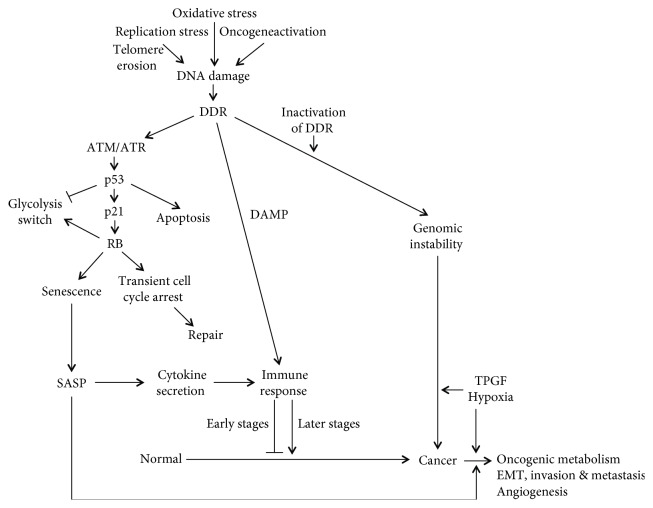
DNA damage-induced senescence plays important roles in cancer development. DNA damage is induced by replication stress/telomere erosion, oxidative stress, and oncogene activation, which activate DNA damage response (DDR). DDR induces transient cell cycle arrest, apoptosis, and cellular senescence. DDR is mediated by checkpoint kinases ATM and ATR, which induce p53. In DDR, p53 induces the expression of many proapoptotic proteins including the BCL2 family (BAX, BID, PUMA, and NOXA) to induce apoptosis. In addition, p53 induces the expression of p21 which in turn activates RB tumor suppressor. p53, p21, and RB are involved in transient cell cycle arrest and cellular senescence. The p53-mediated cell cycle arrest, apoptosis, and cellular senescence act as tumor suppressive processes. Under transient cell cycle arrest, DNA damage is repaired by diverse DNA damage repair mechanisms. However, extensive DNA damage or insufficient repair by inactivation of DDR leads to genomic instability, then tumor-promoting growth factor (TPGF) and hypoxia contribute to cancer development and tumor progression. Senescent cells secrete senescence-associated secretory phenotype (SASP) proteins, consisting of growth factors, immunomodulatory chemokines and cytokines, extracellular matrix- (ECM-) remodeling proteases (matrix metalloproteinases), and ECM/insoluble proteins. The secreted cytokines induce an immune response. In addition, damaged DNA is recognized as a damage-associated molecular pattern (DAMP), triggering an immune response. Immune response inhibits cancer development at early stages, whereas it promotes cancer development at later stages. Furthermore, senescent cells exhibit increased glycolysis, which is regulated by the counterbalance of p53 and RB. Finally, SASP induces EMT, invasion, metastasis, and angiogenesis that are crucial for tumor progression.

Cells that undergo transient cell cycle arrest accurately repair DNA damage before cell cycle progression [168, 173, 174] (Figure 1). However, if the DNA damage is extensive or not effectively repaired by inactivation of DDR and/or checkpoint, the cell is subjected to genomic instability. There are four DNA damage repair mechanisms: homologous recombination (HR), nonhomologous end joining (NHEJ), base- or nucleotide-excision repair pathways (BER or NER), and mismatch repair (MMR). The mutations in DNA repair genes and p53 mutation lead to impairment of DNA damage response pathways leading in turn to continuous formation of DNA DSBs, thereby inducing the genomic instability [167, 168, 170]. Genomic instability drives cancer development in hereditary cancers [174, 179]. Thus, genomic instability and highly frequent p53 mutations are important in oncogene-induced cancer development and progression [168, 173, 174].

If DNA damage is not severe enough to induce genomic instability, it induces senescence as well as transient cell cycle arrest. Senescence has been shown to be a tumor suppressor pathway. In fact, senescence acts as a tumorigenesis barrier in preneoplastic lesions [167, 168, 172, 174]. Recent several studies have demonstrated a significant proportion of senescent cells in many cancers, including B cell lymphoma and lung, breast, colorectal, and thyroid cancers [180–190]. Oncogenic activation-induced senescence has commonly been observed in premalignant tumors; however, it is rare in the malignant counterparts [164, 171, 172, 185, 190, 191].

Interestingly, Snail, ZEB, and Twist confer resistance to senescence and prevent oncogene-induced senescence and replicative senescence in cancers. Snail inhibits oncogene-induced senescence by decreasing p16INK4a expression, thereby helping premalignant cells to escape the oncogene-induced senescence, which acts as a tumorigenesis barrier [192, 193].

Recently, senescence has been implicated in the promotion of tumor progression; thus, senescence exerts a dual function in tumorigenesis. Cellular senescence leads to the secretion of diverse growth factors, cytokines, chemokines, and ECM-remodeling proteases, as a form of the senescent-associated secretory phenotype (SASP) [168, 194–198]. The expression of SASP-associated genes is regulated by several transcriptional factors, including NF-*κ*B, c/EBP*β*, and GATA4. The expression of cell surface–bound IL-1*α* is an early response of senescence. IL-1*α* acts in a juxtacrine manner and binds to the IL-1 receptor, thereby initiating the signal cascade that activates the transcription factors NF-*κ*B and C/EBP*β*. The transcription factors subsequently stimulate the expression of many SASP proteins, including IL-1*α* and the inflammatory cytokines IL-6 and IL-8 [171, 195, 199, 200].

Many factors that compose SASP have numerous biological activities, all highly dependent upon physiological contexts, including the nature of the senescence stimulus, cellular context, and duration and composition of the SASP response. These regulate cell proliferation and induce EMT, angiogenesis, and chronic inflammation, stem cell renewal, and/or differentiation. This suggests that SASP has a dual role (beneficial or detrimental) in tumorigenesis. It can act as tumor suppressor in normal cells or low-grade premalignant cells by inducing aging and otherwise promote tumor progression in high-grade premalignant and malignant cells [171, 199] (Figure 1).

Senescence triggers an immune response. The transcription factors NF-*κ*B and C/EBP*β* stimulate the expression of various cytokines including IL-1*α* and IL-6 and IL-8, thus activating immune response [167, 168, 200]. In addition, extensive DNA damage generates extracellular or extranuclear DNA fragments, which can be detected as a damage-associated molecular pattern (DAMP), thereby triggering immune response [167, 168]. Furthermore, DNA sensor proteins, including the Mre11-Rad50-Nbs1 (MRN) complex and Ku70, recognize DNA damage and then activate NF-*κ*B and IFN-regulatory factor (IRF) response, thus activating immune response [167, 168]. Immune response has a stage-dependent dual role in tumorigenesis. At early stages, immune response removes senescent cells with chronic DNA damage or oncogene activation, to suppress tumorigenesis. The tumor-suppressive effects are performed by attracting and activating immune cells, thereby inducing an innate and adaptive antitumor immune response. SASP recruits and activate T cells and natural killer (NK) cells to the tumor microenvironment, resulting in the elimination of senescent tumor cells in a process termed senescence surveillance. However, at later stages, immune response promotes tumorigenesis through the persistent senescent cells developing a proinflammatory, immunosuppressive microenvironment [167, 168].

Senescent cells also exhibit distinctive metabolic phenotypes, such as increased glycolysis over mitochondrial oxidative phosphorylation. Subsequent studies showed that these metabolic changes are a key characteristic of senescent cells [167, 201–203]. AMP-activated protein kinase (AMPK) is activated in these cells, which in turn activates p53 and RB, resulting in the arrest of cell proliferation. p53 and RB are involved in glycolysis as well as cell cycle arrest. p53 inhibits glycolysis, whereas RB elevates glycolysis; thus, glycolysis in senescent cells may be regulated by the counterbalance of p53 and RB [167, 201, 202]. Senescence-associated metabolic reprogramming has been shown to serve as a target for improving treatment outcomes in a mouse lymphoma model. When glucose utilization was blocked in a murine lymphoma model, chemotherapy-induced senescent tumor cells and their SASP were eliminated, resulting in the inhibition of inflammation and proliferation [204]. Furthermore, recently, senescence-associated reprogramming has been shown to change cancer cells into a stem-like state to avoid a chemotherapy-induced cell-cycle arrest. p53 and H3K9me3 were involved in the acquisition of stem cell-related properties [205].

SASP factors positively modulate tumor development in senescent tumor cells, as well as in high-grade premalignant and malignant cells by regulating all steps of tumor progression, such as facilitating tumor proliferation, inducing EMT, invasion, and metastasis, and indirectly promoting angiogenesis [171, 194–198, 201]. SASP components such as IL-6, IL-8, and MMPs can promote cancer progression. SASP elements exert paracrine effects on the microenvironment as well as the neighboring cells. SASP can remodel the ECM through the activity of ECM-degrading proteases, which modify the structure of the tumor microenvironment, thereby facilitating tumor cell motility, invasion, and metastasis. Senescent osteoblasts can also cause changes in the microenvironment to pave the path for the metastasis of murine breast tumor cells to the bone [194, 199]. Senescent tumor cells exhibit higher rates of invasion by inducing the SASP compared to nonsenescent tumor cells. CXCL12/CXCR4 signaling induces collective invasion and promotes the survival of cancer cells in papillary thyroid carcinoma. Thus, the SASP of senescent cells plays a crucial role in cancer invasion and metastasis [190].

## 3. Regulation of EMT by Oncogenic Metabolism

### 3.1. Oncogenic Metabolism

Most cancer cells rely on a high rate of glycolysis instead of mitochondrial oxidative phosphorylation to produce their energy even under aerobic conditions. This phenomenon is known as the Warburg effect, aerobic glycolysis, or the glycolytic switch [206–214]. The Warburg effect has long been considered the dominant metabolic phenotype of cancer cells. Cancer cells display diminished oxidative phosphorylation due to mitochondrial dysfunction. However, recent studies have suggested that mitochondria in most tumor cells are not defective in oxidative phosphorylation (OXPHOS). Mitochondrial function and OXPHOS have become recognized as being an important role in tumorigenensis, metastasis, and cancer stemness in cancer cells [215–221].

Cancer cells also exhibit elevations of other oncogenic metabolic pathways, including glutamine metabolism, pentose phosphate pathway, and synthesis of fatty acids and cholesterol. Alterations of cellular metabolism in cancer cells produce intermediate biosynthetic precursors for nucleic acids, lipids, and proteins [208–214, 222–224]. These alterations also confer many advantages for survival and proliferation in cancer cells. These metabolic changes are mediated by cancer-related transcription factors, including HIF, c-Myc, and p53, and are actively regulated by various cancer-related signaling, such as PI3K and AMPK pathways [214, 225].

Tumor cells in hypoxic regions of the tumor consume glucose and release lactate in glycolytic metabolism. Oxygenated cancer cells consume the lactate released by hypoxic cancer cells to produce ATP through oxidative energy production. Thus, glycolytic and oxidative tumor cells mutually regulate their activities concerning energy metabolites in a process of metabolic symbiosis [226–228]. Although the tricarboxylic acid (TCA) cycle stalls in cancer cells, mitochondria actively exhibit OXPHOS.

CSCs share general features of metabolic processes with non-stem cancer cells [229, 230]. Metabolic reprogramming is important in CSC biology. The presence of glucose in the microenvironment contributes to the increased fraction of stem-like cancer cells in tumors, whereas glucose starvation rapidly depletes stem-like cancer cells. These phenomena are related with the enhanced glucose metabolism pathway of CSCs [230, 231].

CSCs exhibit higher glucose uptake, lactate production, glycolytic enzyme expression, and ATP content than do non-stem cancer cells. The stemness marker CD44 also contributes to the regulation of glycolytic metabolism [232]. CSCs of glioblastoma that are very reliant on glycolysis contribute to migration in hypoxic conditions. Therefore, glycolytic metabolic reprogramming is involved in the maintenance of CSCs and cancer progression.

Recently, it was reported that the quiescent or slow-cycling tumor-initiating CSCs are highly dependent on OXPHOS when compared with the differentiated cancer progeny cells in many other tumor types. It implies that CSCs preferentially use mitochondrial oxidative metabolism for glycolysis [230, 233–236].

Furthermore, CSCs display increased mitochondrial mass and membrane potential and increased oxygen consumption rates. Invasive cancer cells induce very elevated mitochondrial metabolism by increasing the expression of the transcription factor peroxisome proliferator-activated receptor gamma coactivator (PGC)1*α*, which is a master regulator of mitochondrial biogenesis [237, 238].

CSCs may exhibit increased glycolysis or increased OXPHOS depending on the cancer type. The metabolic phenotype of the CSCs is fine-tuned between glycolysis and OXPHOS by the environment and cellular signaling pathway. The CSC metabolic phenotype can switch to glycolysis during the inhibition of OXPHOS [230, 239, 240].

Since the metabolic pathways are connected with each other in complicated manners, glycolysis and OXPHOS alone cannot be considered as the basis of CSC metabolism. CSCs rely on the metabolism of glucose and glutamine. CSCs utilize glutamine metabolism for the biosynthesis of amino acids, nucleotides, and lipids because glutamine provides carbon and amino-nitrogen [241]. Thus, glutamine metabolism is closely linked to glucose metabolism in CSC metabolism. Additionally, CSCs also rely on lipid metabolism to increase their bioenergetic requirements. The lipid metabolism is also closely linked in tumor metastasis [242].

However, mitochondrial function does play an important role in either case and contributes to CSC functions, including stemness, migration, and drug resistance [230]. NANOG is a stem cell marker. Functionally, the protein regulates metabolic reprogramming by inhibiting mitochondrial OXPHOS and activation of fatty acid oxidation, thereby promoting self-renewal, tumor initiation, and chemoresistance of tumor-initiating stem-like cells [243].

In CSCs, the capacity for chemoresistance is involved in the increased OXPHOS phenotype and expression of PGC1*α* [244–247]. Cells that survive chemotherapy can exhibit enhanced mitochondrial OXPHOS. MYC and MCL1 increase mitochondrial OXPHOS and ROS, contributing to chemotherapy-resistant CSCs in TNBC [247].

In addition, it has very recently been reported that the metabolic phenotype plasticity of pancreatic CSCs is determined by the balance between MYC and PGC-1*α* [235]. The microenvironment surrounding CSCs contributes to maintenance of stemness. The different microenvironments, including different oxygen tension and glucose concentrations, can induce different cancer phenotypes in diverse tissues. For example, in glucose-rich environments, CSCs rely on aerobic glycolysis for their energy production and cell proliferation, whereas in glucose-deprived conditions quiescent CSCs utilize the mitochondrial oxidative metabolism to generate ATP. Under hypoxia, CSCs increase the expression of the glucose metabolic enzymes and then shift to a more glycolytic phenotype to adapt to this environment [240]. In normal stem cells and induced pluripotent stem cells, OCT4, KLF4, SOX2, and MYC are involved in the glycolytic metabolism phenotype and stemness [233]. However, the stemness of CSCs is maintained by NOTCH, WNT/*β*-catenin, PI3K/Akt, phosphatase and tensin homolog (PTEN), NF-*κ*B, KRAS, HIF, TP53, and many oncogenic pathways. The metabolic phenotype of CSCs is also affected by these signals [230]. Targeting metabolic reprogramming may eliminate CSCs with potentially significant benefits in cancer treatment.

The tumor microenvironment is also associated with neoplastic mitochondria. The metabolism of tumor stroma cells, such as cancer-associated fibroblasts (CAF), is reprogrammed. CAF, which is the key component of tumor stroma that surrounds the tumor, represent aerobic glycolysis, like tumor cells. CAF secrete energy metabolites, such as lactate and pyruvate, generated by anabolic glycolysis. Cancer cells assimilate lactate from CAF and utilize mitochondrial OXPHOS for efficient energy production, resulting in a higher proliferative capacity. It is the reverse Warburg effect. Emerging evidence supports the idea that cancer cells exhibit a hybrid glycolysis/OXPHOS phenotype, which plays an important role in energy production and biomass synthesis by cancer cells. The hybrid glycolysis/OXPHOS phenotype leads to enhanced metabolic plasticity of cancer cells for better survival in response to external stimuli and contributes to metastasis and therapeutic resistance. In addition, CAF secrete various cytokines and a distinctive ECM, which promotes tumor growth, invasion, and tumor progression [215, 220, 248–251].

### 3.2. Regulation of Oncogenic Metabolism by EMT-Inducing Transcription Factors

**Figure 2:**
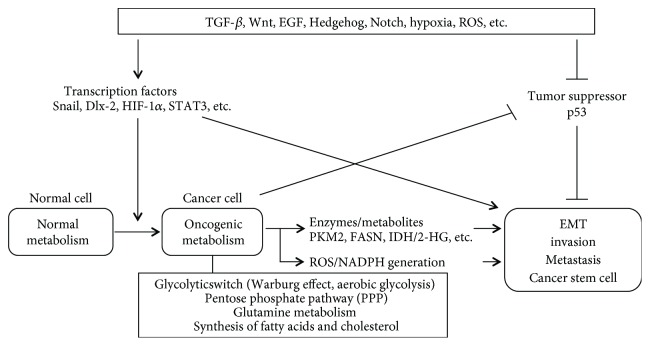
Oncogenic metabolism plays an important role in the regulation of EMT. Four main mechanisms are involved in oncogenic metabolism-induced EMT: (1) regulation of both EMT and oncogenic metabolism by several transcription factors, (2) regulation of EMT by oncogenic enzymes and metabolites, (3) negative regulation of p53 by oncogenic metabolism, and (4) regulation of ROS and NADPH generation by oncogenic metabolism. (1) Several transcription factors and signaling factors involved in EMT or CSCs, including Snail, Dlx-2, HIF-1*α*, STAT3, TGF-*β*, Wnt, EGF, Notch, Hedgehog, hypoxia, and ROS, have been shown to regulate oncogenic metabolism. (2) PKM2 is translocated to the nucleus and then directly interacts with TGF-*β*-induced factor homeobox 2 (TGIF2) in colon cancer cells, thereby recruiting histone deacetylase 3 (HDAC3) to suppress E-cadherin transcription. PKM2 can also enhance tumor migration via PI3K/Akt signaling in gastric cancer. A FASN-TGF-*β*1-FASN-positive loop leads to high EMT/metastatic potential in cisplatin-resistant cancer cells. Furthermore, oncometabolites contribute induction of EMT. IDH1/2 mutations that induce the accumulation of 2-HG lead to EMT by ZEB1 upregulation and miR-200 downregulation in breast tumors and in colorectal cancer cells. The high levels of D-2-HG positively regulate the expression of ZEB1 in colorectal cancer cell, thereby inducing EMT. (3) Glutamine metabolism has been shown to downregulate p53 levels. Glutamine metabolism inhibition increases p53 expression and then induces the p53-dependent upregulation of Snail-targeting microRNAs, thereby leading to EMT induction by decreasing Snail mRNA levels. In addition, the abnormal TCA cycle enzymes, such as IDH mutants, prevent p53 activities. (4) Mutations of IDH, SDH, and FH induce ROS production. ROS play an important role in EMT. IDH mutation inhibits the production of NADPH and increases the consumption of NADPH. FH mutation also decreases NADPH levels by accumulation of fumarate. NADPH homeostasis plays an important role in the survival of cancer cells.

EMT can regulate the metabolic reprogramming of cancer cells by regulating the many regulatory molecules involved in EMT, including Snail, Dlx-2, HIF-1*α*, STAT3, TGF-*β*, Wnt, and Akt. The induction of EMT contributes to the acquisition of CSC properties and also leads to the repression of mitochondrial metabolism and induction of the glycolytic switch [39, 40, 206, 240, 252–259] (Figure 2).

Snail has recently been shown to promote metabolic alterations [39, 40, 206, 240, 254, 260]. Snail leads to the downregulation of cytochrome C oxidase (COX) subunits or fructose-1,6-bisphosphatase 1 (FBP1), resulting in mitochondrial repression and promotion of glucose metabolism [39, 40, 206, 240, 254]. Snail also controls glucose flux by suppressing the phosphofructokinase platelet (PFKP) under metabolic stress. PFKP is a major isoform of cancer-specific phosphofructokinase-1 (PFK-1), which is linked to the first rate-limiting step of glycolysis. The inhibition of PFKP switches the glucose flux toward the pentose phosphate pathway, resulting in NADPH production [260].

Dlx-2 plays a critical role in EMT and the glycolytic switch [22, 39, 40]. Dlx-2 expression is induced by the metabolic stress-dependent induction of ROS and may contribute to tumor progression through the regulation of metabolic stress-induced necrosis [261]. Recently, it was demonstrated that Dlx-2 plays an important role in the TGF-*β*/Wnt-induced glycolytic switch and mitochondrial repression by increasing Snail expression [39]. TGF-*β*/Wnt inhibits mitochondrial complex IV (i.e., COX) to prevent mitochondrial respiration [39, 40]. In addition, canonical Wnt signaling promotes glycolysis through the upregulation of pyruvate dehydrogenase (PDK), Myc, and monocarboxylate transporter 1 (MCT-1) [256–258]. Dlx-2 also induces the expression of glutaminase (GLS), a glutamine metabolism enzyme, and the Dlx-2/GLS1/Gln metabolism axis contributes to the TGF-*β*/Wnt-induced, Snail-dependent EMT, and glycolytic switch [40].

HIF-1 acts as a major regulator of glycolytic enzymes, including GLUT, hexokinase, lactate dehydrogenase (LDH), and MCT, thereby contributing to the glycolytic switch [252, 253, 262]. Furthermore, HIF-1 negatively regulates mitochondrial function and oxygen consumption by inducing pyruvate dehydrogenase kinase (PDK), which inhibits pyruvate dehydrogenase (PDH), thereby preventing the flow of pyruvate into the TCA cycle [252, 253].

In addition, the positive feedback loop between HIF-1, STAT3, and pyruvate kinase M2 (PKM2) contributes to the induction of proteins involved in glycolysis [263–266]. Oxygen deprivation, growth factors, or oncogene-induced HIF-1*α* enhances the translation of PKM2, which is an isoform of pyruvate kinase, a rate-limiting enzyme of glycolysis. On the other hand, cytokines, growth factors, or oncogene-induced STAT3 activation leads to HIF-1*α* transcription and STAT3 activation, which also induces a PKM2/HIF-1*α*-positive feedback loop [263–266].

STAT3 also contributes to the EMT-induced metabolic changes. Mammosphere culture produces stable EMT cells in epithelial breast cancer cells. STAT3 is activated in these EMT-derived cancer cells, which induces aerobic glycolysis and the upregulation of certain enzymes and transporters related to glycolysis (such as MCT2). Inhibition of STAT3 prevents EMT-associated changes with the overexpression of MCT2 and ZEB1 in EMT-derived cancer cell lines. This suggests that STAT3 is required for the EMT-induced metabolic changes [255].

Akt has important roles in the glycolytic switch and invasiveness in cancer cells. Overexpression of Akt promotes glycolytic metabolism and mitochondrial dysfunction. It also switches from a radial growth (i.e., noninvasive) melanoma to vertical growth (i.e., invasive) melanoma [259].

### 3.3. Regulation of EMT, Metastasis, and Stemness by Oncogenic Metabolic Enzymes

Several metabolic enzymes, including pyruvate kinase M2 (PKM2), LDH, pyruvate carboxylase (PC), fatty acid synthase (FASN), aldolase, glutaminase (GLS), citrate synthase (CS), and fructose-1,6-bisphosphate (FBP), have been associated with EMT, metastasis, and CSCs [22, 40, 240, 267–285] (Figure 2).

Glucose metabolism has been linked with EMT and CSCs [22, 240, 267, 270, 276, 278, 279]. PKM2 decreases oxygen consumption and enhances glucose uptake and lactate production in cancer cells and also leads to the accumulation of products of macromolecular biosynthesis by promoting anabolic metabolism and growth of cancer cells [264, 275]. PKM2 is translocated to the nucleus in response to EMT-inducing stimuli. PKM2 then directly interacts with TGF-*β*-induced factor homeobox 2 (TGIF2), a transcriptional repressor of TGF-*β* signaling, and suppresses E-cadherin transcription by recruiting histone deacetylase 3 to promote EMT [22, 267]. PKM2 also enhances the ability of tumor migration by inducing the PI3K/Akt signaling pathway in gastric cancer [276].

High levels of LDH A trigger the expression of EMT and CSC markers in invasive bladder cell lines and in muscle-invasive bladder cancer specimens. Thus, LDH A likely has a critical role in the activation of EMT and CSCs [22, 270].

Silencing of the glycolytic enzyme aldolase A suppresses cell proliferation, invasion, and the EMT phenotype in colon cancer. The mRNA and protein levels of aldolase A are upregulated during the progression of human colon cancer and high aldolase A protein expression promotes EMT and migration of colon cancer cells. In addition, aldolase A interacts with HIF-1 and other EMT-related signaling pathways and affects the development of colon cancer. Thus, aldolase A contributes to tumor progression by inducing EMT and is correlated with poor prognoses in colon cancer [278, 279].

In addition, loss of FBP, the gluconeogenesis regulatory enzyme, is important in the EMT-driven CSC phenotype. The expression of FBP1 is suppressed by Snail. Snail also enhances glycolysis, suppresses oxygen consumption and ROS production, and promotes EMT and CSC phenotypes by inducing epigenetic silencing of FBP1 [22, 240].

The dysregulation of lipid metabolism and glutaminolysis has also been associated with EMT [22, 271, 277]. FASN signaling plays important roles in determining the epithelial or mesenchymal state of a cell to modulate subcellular structural components. In stem-like cells, transient knockdown of FASN inhibits structural hallmarks of EMT. Loss of FASN signaling leads to a stable tumor reversion for a normal-like tissue phenotype and also prevents the tumorigenicity of metastatic breast cancer cells in vivo [277]. In addition, FASN induces the upregulation of TGF-*β* levels and TGF-*β* also leads to the induction of FASN expression. A FASN-TGF-*β*1-FASN regulatory loop is involved in high EMT/metastatic potential in cisplatin-resistant cancer cells [271].

Glutaminase 1 (GLS1) is the first enzyme in glutamine anaplerosis. GLS1 promotes tumor growth and metastasis [22, 40]. The expression of GLS1 is enhanced in breast and prostate cancers and HCC tissues, compared to normal tissues [280, 281], in which Myc participates [282, 283]. GLS1 is induced by Dlx-2 and also by TGF/Wnt in a Dlx-2-dependent manner. Dlx-2-, TGF-*β*-, Wnt-, and Snail-induced EMT and the glycolytic switch are prevented through the inhibition of glutamine metabolism by short hairpin GLS1, Gln deprivation, and Gln metabolism inhibitors. These results indicate that the Dlx-2/GLS1/glutamine metabolic axis is a crucial regulator of TGF-*β*/Wnt-induced, Snail-dependent EMT, metastasis, and the glycolytic switch [22, 40].

In addition, TCA cycle enzymes contribute to EMT and cell migration and invasion [22, 269, 272]. PC in the TCA cycle has been linked to cell migration and invasion. Knockdown of the enzyme suppresses proliferation, migration, and invasion behaviors in invasive breast cancer cells. The overexpression of PC enhances the proliferation, migration, and invasion of noninvasive breast cancer cells [22, 269].

Loss of the TCA cycle enzyme CS is involved in EMT and the glycolytic switch. Knockdown of the enzyme increases the upregulation of Snail and Twist and downregulates p53 and its target genes (TIGAR and SCO2), resulting in EMT, mitochondrial repression, and the glycolytic switch. p53 prevents glycolysis by increasing the expression of TIGAR and enhances mitochondrial respiration by the activation of SCO2 [22, 272]. Thus, many oncogenic metabolic pathways may be interconnected in cancer cells and are also closely related with EMT and metastasis. Inhibition of any component enzyme in the overall oncogenic metabolism may influence the inhibition of cancer cell metastasis.

The Warburg effect leads to the destruction of the ECM and induces metastasis by the induction of an acidic environment [209, 286]. A glycolytic mechanism also plays an important role in regulation of the angiogenic switch that effects metastatic growth [22, 287]. In addition, fluorine-18 fluorodeoxyglucose uptake in TNBC is significantly higher than in tumors positive for estrogen receptor and progesterone receptor and negative for human epidermal growth factor receptor. Increased glycolysis in cancer is probably associated with aggressiveness [288].

Metabolic reprogramming toward aerobic glycolysis unavoidably induces the accumulation of potent toxic metabolites, such as reactive carbonyl species. Methylglyoxal, a reactive dicarbonyl intermediate, is formed as a side product during glycolysis in cancer cells. Methylglyoxal accumulation correlates with tumor growth and metastasis [289, 290]. Methylglyoxal is able to modify proteins, lipids, and nucleotides and leads to the cellular dysfunction and mutagenicity [289]. The mediated glycation of specific target proteins results in a protumorigenic potential and promotes tumor progression. In cancer cells, methylglyoxal leads to induced tumor growth and metastatic potential in vivo [290].

High levels of methylglyoxal maintain the nuclear localization and activity of Yes-associated protein (YAP) in breast cancer cells. YAP is a key transcriptional coactivator that is involved in the induction of tumor growth and invasion and contributes to cancer progression by transcriptional activation of c-myc and CTGF [290]. Recently, YAP activation has been shown to be involved in glucose deprivation stress and aerobic glycolysis [291–293]. In addition, methylglyoxal induces YAP nuclear accumulation and leads to YAP cotranscriptional activity in breast cancer cells [290]. The glycolysis-induced methylglyoxal stress also regulates the expressions of key glycolytic enzymes by YAP activity [290, 294].

Besides metabolic enzymes and metabolites, proteins involved in cell survival, including heat shock protein (Hsp)90 and Hsp27, are associated with metabolic alterations and EMT. The Hsp90 molecular chaperone is important in cell proliferation, cell cycle progression, and apoptosis. Hsp90 also functions as an oncogenic protein to regulate metabolic alterations, EMT, invasion, metastasis, and drug resistance [295–303].

Hsp90 also contributes to induction of proliferation, glycolysis, and inhibition of apoptosis by phosphorylation of PKM2 at the Thr-328 residue [299]. Hsp90 induces this phosphorylation in a GSK-3*β*-dependent manner. The phosphorylation stabilizes PKM2 and contributes to biological functions that include regulation of glycolysis, mitochondria respiration, cell apoptosis, proliferation, and cofactor function. Furthermore, the expression of HSP90 positively regulates PKM2 expression in HCC tissues and overexpression of HSP90 and PKM2 are associated with poor prognosis of HCC patients [299]. Inhibition of Hsp90 also suppresses EMT, invasion, and motility by downregulating HIF-1*α* and NF-*κ*B [303].

Hsp90 is secreted by tumor cells. Extracellular Hsp90 interacts in an autocrine or paracrine manner with the surfaces of adjacent cells to enhance the growth and metastasis [304]. Extracellular Hsp90 promotes cell motility and invasion in cancer cells and metastasis in preclinical models. In prostate cancer, extracellular Hsp90 functions as a novel regulator of EMT. Secretion of Hsp90 b from tumor cells induces the phosphorylation of extracellular signal-regulated kinase by receptor low-density lipoprotein-related protein, and signaling by the complex of extracellular Hsp90, low-density lipoprotein-related protein 1, and extracellular signal-regulated kinase promotes cell motility and EMT by inducing matrix metalloproteinases and several EMT transcription factors, including Snail, Twist, Zeb, and Slug [305].

Hsp90 is associated with methylglyoxal-mediated metastasis. Methylglyoxal also induces the downregulation of Hsp90 expression levels in human retinal pigment epithelial cells [306]. Hsp90 regulates the stabilization and activation of numerous oncoproteins called Hsp90 clients, which play an important role in cellular signal transduction pathways and adaptive responses to stress [295]. The large tumor suppressor 1 (LATS1) is one client of the Hsp90 chaperone protein. In the Hippo pathway, which plays a pivotal role in tissue homeostasis, LATS1 induces antiproliferative signals by inhibiting the nuclear translocation and oncogenic activity of YAP [290, 294]. Hsp90 regulates LATS1 kinase expression level and activity. The downregulation of LATS1 mRNA expression by promoter hypermethylation contributes to the aggressive breast cancer phenotype and is correlated with poor survival [307]. Methylglyoxal leads to decreased LATS1 expression by proteasome degradation, thereby sustaining the activity of YAP in the nucleus. Methylglyoxal also enhances Hsp90 posttranslational glycation, leading to LATS1 degradation. Therefore, the increased glycolytic rate in tumor cells unavoidably leads to the accumulation of potent glycating agents, such as methylglyoxal, which in turn induces YAP nuclear persistence and activity by the inhibition of Hsp90 and subsequent decrease of LATS1 kinase. These changes result in YAP-mediated tumor growth and metastasis [290].

Hsp27 is another small molecular chaperone. It contributes to the malignant properties of cancer cells, including EMT and drug resistance. It is avidly expressed in aggressive cancers. Hsp27 knockdown induces proteasomal degradation of Snail, thereby suppressing TGF-*β*1-induced EMT. Thus, Hsp27 induces EMT by inducing Snail stabilization [308, 309]. Hsp27 also contributes to the maintenance of CSC by regulating the EMT process and NF-*κ*B activity in breast cancer [309].

### 3.4. Regulation of EMT/Metastasis by Mitochondrial Metabolism

#### 3.4.1. Oncogenic Dysfunction of Mitochondria

Mitochondria have important roles in cellular functions that include energy production, apoptosis, control of cytosolic Ca^2+^ levels, synthesis of macromolecules, generation of metabolites for epigenetic regulation, and the innate immune response [310–316]. Mitochondria also provide several metabolites, including NAD^+^, ATP, *α*-ketoglutarate, and acetyl CoA, which are required for many transcriptional and epigenetic processes, including chromatin remodeling, histone modifications, and nucleosome positioning [217, 314, 317, 318].

Contrary to the conventional wisdom that mitochondria are not functional in cancer cells, functional mitochondria are in fact essential for cancer cells. Although mutations in mitochondrial genes are common in cancer cells, they do not inactivate mitochondrial energy metabolism, but rather alter the mitochondrial bioenergetic and biosynthetic state. The mitochondrial dysfunction is implicated in the metabolic reprogramming of cancer cells. The dysregulation of mitochondrial respiration may enhance glycolysis in cancer cells [218, 312, 319–321].

Mitochondrial DNA alterations are commonly caused by point mutations and copy number changes in cancer cells, which contributes to cancer progression by increasing the chemoresistance or invasive phenotype. For example, mitochondrial DNA mutations induce the complex I defect, which is accompanied by the overproduction of ROS and upregulation of nuclear genes essential for cell survival and angiogenesis, which promotes the metastatic potential of tumor cells [312]. In addition, mutations of mitochondrial DNA can protect cancer cells from stress-induced cell death by activating the PI3/Akt pathway and therapeutic agents [312].

Mitochondrial oxidative stress can enhance tumor progression and the metastatic potential of cancer cells [312]. Mitochondria act in ROS signaling and sensing [314]. Mitochondria can alter energy states in the chemical environment of a cell and can lead to the alternation of the levels of endogenous metabolites, including iron (II), succinate, and ascorbate, as well as various forms of ROS by acting as a redox sensor [314]. Mitochondria induce the direct provision of substrates and affect epigenetic signaling indirectly through the generation of ROS [314, 322, 323].

In response to ROS, epigenetic alterations may contribute to the regulation of mitochondrial metabolism by inducing the altered expressions of genes. In addition, alterations of epigenetic patterns, including global DNA methylation and histone modifications, are induced by endogenous metabolite levels, metals, and other environmental pollutants in vitro and in vivo [314, 324, 325].

#### 3.4.2. Regulation of EMT by Mitochondrial Metabolism

Mitochondria have important roles in the alteration of metabolic processes in tumors [321]. Alterations of mitochondrial metabolism are induced by the inactivation of components of the TCA cycle and electron transport chain [326–330]. Deregulated cellular energetics in cancer cells may be induced by defection of mitochondrial metabolic enzymes, including fumarate hydratase (FH), succinate dehydrogenase (SDH), and isocitrate dehydrogenase (IDH) [319].

SDH and FH act as tumor suppressors. However, mutations of SDH and FH induce the accumulation of succinate or fumarate, respectively, in tumors and leads to EMT, metastasis, and tumorigenesis [331–333]. Genetic alterations of FH, SDH, and IDH are closely associated with EMT. Such mutations have been observed in various cancers. In fact, genetic alterations of mitochondrial metabolic enzymes lead to the accumulation of mitochondrial metabolites, which contribute to the activation of the oncogenic signaling cascade in tumors [19, 225, 319, 334].

SDH is a heterotetrameric and highly conserved protein. SDH consists of catalytic subunits (SDHA and SDHB) and binding sites for ubiquinones (SDHC and SDHD) [335]. SDHB mutations are often present in malignant and metastatic tumors. Knockdown of SDHB induces the altered use of glucose and glutamine and leads to mitochondrial dysfunction, thereby inducing EMT [331, 332]. SDHB mutations also activate EMT-inducing transcription factors, such as Snail and Slug, and lead to invasive human metastatic pheochromocytomas and paragangliomas, indicating that SDHB mutations are linked to the induction of EMT in these tumors [336]. In chromaffin cells, loss of SDHB leads to the induction of EMT-inducing transcription factors and to the epigenetic silencing of keratin-19, a component of the intermediate filaments, which is involved in aggressive behavior characterized by enhanced migratory, adhesive, and invasive properties [337, 338]. In addition, SDHB deficiency enhances cell migration and invasion by regulating the TGF*β*/Snail-mediated process in colorectal cancer and ovarian cancer [332, 339]. Loss of the assembly factor SDH5 in lung cancer cells induces EMT, and SDH mutation also induces the activation of the GSK-3*β*/*β*-catenin axis to induce metastasis in vivo [338, 340]. SDHB deficiency in epithelial kidney cells leads to the accumulation of succinate, which acts as a mediator of EMT. Succinate accumulation induces EMT by inhibiting miR-200 [19].

FH deficiency is associated with a highly aggressive phenotype that is prone to metastasize and has been correlated with a very poor clinical outcome in renal cancer. In addition, fumarate accumulation caused by the loss of FH can be critical in transformation. Loss of FH and increased fumarate accumulation are closely associated with the induction of EMT in mouse and human cells. Fumarate accumulation elicits EMT by epigenetic suppression of the miR-200 antimetastatic miRNA and suppresses several transcription factors, including Slug and ZEB1/2. Loss of FH and fumarate accumulation may play a critical role in aggressive features of cancer cells [333, 341, 342].

IDH is another mitochondrial enzyme involved in EMT. IDH catalyzes the oxidative decarboxylation of isocitrate to *α*-ketoglutarate. The gain-of-function IDH1/2 mutations convert *α*-ketoglutarate to 2-hydroxyglutarate, which may contribute to the inhibition of DNA demethylases and aberrant regulation of gene expression patterns. IDH1/2 mutation leads to formation of 2-hydroxyglutarate in acute myeloid leukemia and glioblastoma. High levels of 2-hydroxyglutarate have frequently been observed in tumors harboring IDH mutations. These metabolites dysregulate the competitive inhibition of *α*-ketoglutarate–dependent processes, including demethylation of histones [314, 343, 344]. IDH1/2 mutations that induce the accumulation of 2-HG lead to EMT by ZEB1 upregulation and miR-200 downregulation in breast tumors and in colorectal cancer cells [345]. The high levels of 2-hydroxyglutarate in colorectal cancer specimens are also associated with metastasis [346].

SDH or FH mutation induces the stabilization of HIF-1*α* by accumulation of succinate and fumarate which prevent HIF-*α* prolyl hydroxylation [347–350]. Succinates accumulated by SDH mutation are exported from mitochondria and then inhibit the activity of HIF-*α* prolyl hydroxylase, thereby inducing the stabilization of HIF-1*α* [335, 347, 351]. Reduced FH activity also leads to HIF-1*α* stabilization by accumulation of fumarate in FH mutant cells and then a shift in metabolism from oxidative phosphorylation to glycolysis [335, 348–350].

In addition, IDH1 mutation also leads to HIF-1*α* stabilization by inhibition of prolyl hydroxylase, which hydroxylates HIF-1*α* and uses *α*-ketoglutarate as a substrate allowing for the proteasomal degradation of HIF-1*α* [347, 351, 352]. Mutation in IDH1 produces 2-hydroxyglutarate, which acts as a competitive inhibitor of prolyl hydroxylase and occupies the *α*-ketoglutarate-binding site on prolyl hydroxylase [335, 352]. These results indicate that the defects of the mitochondrial enzymes SDH, FH, and IDH lead to mitochondrial dysfunction and contribute to cancer progression.

PGC-1*α* is a master integrator of cellular signals and mediates mitochondrial biogenesis and OXPHOS. It is involved in metastatic dissemination in breast cancer [237]. In addition, silencing of family with sequence similarity 210 member B (FAM210B), a mitochondria outer membrane protein, is closely linked to metastasis and survival in vivo and in vitro. Loss of FAM210B increases mitochondrial respiratory and reduces glycolysis by downregulating pyruvate dehydrogenase kinase 4 and promotes malignant metastasis [353].

Mitochondrial dysfunction is caused by defects in mitophagy. Accumulation results in excessive ROS production [218, 354–356]. ROS promotes metastasis by activating several signal transduction cascades, including SRC and protein tyrosine kinase 2 beta signaling [218, 357]. A genetic signature of mitochondrial dysfunction is a hallmark of cancer and has been associated with enhanced metastatic dissemination and a dismal prognosis [358]. Imbalances in mitochondrial dynamics are involved in mild ROS overproduction and can lead to increased metastasis [359, 360].

Altered oncogenes/tumor suppressors including HIF-1 and p53 modulate the expression of their target genes to regulate mitochondrial respiration and cellular metabolism. Therefore, mitochondrial dysfunction is crucial for cancer progression. A better understanding of the molecular mechanisms of mitochondrial alterations and mitochondrial retrograde signaling is required to improve the efficacy of selective anticancer therapy [319].

### 3.5. Regulation of Oncogenic Metabolism by p53

Metabolic reprogramming contributes to cell survival and sustains the increased demands of cell proliferation. The activation of oncogenes or the loss of tumor suppressors affects metabolic reprogramming [361]. The tumor suppressor p53 has been closely associated with the metabolic network. p53 reduces anabolic metabolism and preserves cellular energy in response to nutrient stress [361]. p53 directly regulates several metabolic enzymes, including SCO2 [362], GLS2 [72, 363], GLUT1, GLUT4 [364], GLUT3 [365], ME1, ME2 [366], G6PD [361, 367], and PANK1 [368, 369].

p53 mediates mitochondrial respiration by inducing the expression of SCO2 and GLS2. SCO2 is a crucial regulator of the assembly of complex IV, and it is important in OXPHOS. Mitochondrial respiration is stipulated by p53 via the transactivation of SCO2. The glutamine metabolic enzyme GLS2 is important in the TCA cycle and mitochondrial oxidative phosphorylation. The induction of GLS2 by p53 leads to decreased ROS levels due to the increased glutathione (GSH) levels, which protects cells from DNA damage or oxidative stress [72, 362, 363].

p53 also reduces glucose uptake and glycolysis by repressing the expression of glucose transporter (GLUT) 1 and 4 [364]. Loss of p53 increases the rate of aerobic glycolysis through NF-*κ*B-dependent upregulation of GLUT3 [365]. p53 inhibits glycolysis by increasing TIGAR expression, which decreases the intracellular levels of F2 and 6BP and deactivates phosphofructokinase 1, thus reducing glycolysis in cancer cells [370].

Furthermore, p53 inhibits cancer metabolism by activating the AMPK and PTEN energy-sensing mechanisms during nutrient stresses. AMPK*β*1 and PTEN negatively regulate the Akt/PI3K/mTOR signaling pathways, which mediates cell growth, protein translation, autophagy, metabolism, and cell survival. As well, p53 inhibits the Warburg effect by repressing glycolysis and reducing cell growth by acting on AMPK and PTEN [371].

In another function, p53 regulates cell metabolism and proliferation by suppressing the expression of malic enzymes 1 and 2. These enzymes influence NAPDH production, glutamine metabolism, and lipogenesis [361]. p53 also negatively regulates the pentose phosphate pathway. Inhibition of the pathway reduces glucose consumption, NADPH production, and biosynthesis. p53 directly binds to glucose-6-phosphate dehydrogenase, the first enzyme of the pentose phosphate pathway, and inhibits the enzyme's activity, thereby functionally blocking the pathway [361, 367]. Pantothenate kinase 1 is a target of p53. This kinase catalyzes the rate-limiting step for CoA synthesis. p53 promotes gluconeogenesis by elevating pantothenate kinase 1 expression in the liver [368, 369, 372]. Thus, p53 appears to affect metabolic reprogramming by regulating several metabolic enzymes.

In still another function, p53 has an important role in anoikis, which contributes to the suppression of metastasis [80, 373]. Anoikis is a type of p53-dependent apoptosis caused by inadequate/inappropriate cell matrix interactions. Inhibition of p53 prevents anoikis in thyroid epithelial cells [374]. Anoikis also acts as an important barrier to metastasis. Tumor cells that acquire malignant potential also acquire anoikis resistance, thereby contributing to metastasis to distant organs [80, 375].

Salt-inducible kinase 1 is a member of the AMPK-related kinase family. The kinase induces the p53-dependent suppression of tumor metastasis by mediating liver kinase B, which is a tumor suppressor that acts as an upstream regulator to activate AMPK. Salt-inducible kinase 1 acts as an upstream regulator of p53-mediated anoikis and leads to p53-dependent anoikis and suppression of metastasis by regulating liver kinase B1 [80, 373].

Inadequate/inappropriate matrix attachment also leads to the production of ROS, which is associated with anoikis. The Warburg effect allows cancer cells to avoid excessive ROS produced by mitochondrial respiration, which contributes to the acquisition of anoikis resistance. This is a survival advantage for metastasis. Consistent with this, p53 tumor suppressor enhances mitochondrial oxidation, whereas HIF and Snail pro-metastatic transcription factors lead to decreased oxidative metabolism [206].

## 4. Mechanism for EMT Regulation by Oncogenic Metabolism

The oncogenic metabolism is important in the induction of EMT [22]. In this process, p53, ROS, and NADPH are crucial in EMT that is mediated by the oncogenic metabolism [40, 335] (Figure 2).

### 4.1. Inactivation of p53 by Oncogenic Metabolism

Accumulating evidence suggests that oncogenic metabolism may negatively regulate p53 [40, 376]. The normal functions of p53 are closely linked to the regulation of p53 stability [377–380]. The ubiquitin-proteasome pathway dominantly regulates p53 stability, localization, and functions. Selective E3 ubiquitin ligases, such as Mdm2, act as the master regulator of the p53 protein levels and activities. In normal cells, Mdm2 maintains the basal level of p53 protein by regulating its ubiquitination and degradation. Under exogenous and endogenous stresses, the Mdm2-p53 binding affinity is significantly decreased, which inhibits p53 degradation [377, 378]. Ubiquitination factor E4B is an E3 and E4 ubiquitin ligase that physically interacts with p53 and promotes Mdm2-mediated p53 polyubiquitination and degradation, thereby inhibiting p53 functions [379].

p53 stability is regulated by a variety of deubiquitinating enzymes that directly or indirectly affect the ubiquitination of p53. These enzymes can regulate various cellular processes associated with p53 and affect diseases, such as cancer [377]. Therefore, we propose that oncogenic metabolism regulates the ubiquitin-proteasome pathway and/or deubiquitinating enzymes to inhibit the activity of p53, which may be correlated with EMT.

In addition, ncRNAs have been suggested to play important roles in p53 dysregulation and tumorigenesis [381]. Recent data revealed the miRNA-mediated regulation of the level and function of p53 and its network [71, 95]. The involved miRNAs interact with critical pathways, including p53, NF-kappa B, and *β*-catenin pathways, and then regulate most physiological and pathological processes, including metastatic tumor progression, EMT, and CSC phenotype [382]. miRNAs mediate the cross-talk between cancer cells and stromal cells, leading to metabolic reprogramming [383].

Specific miRNAs directly target p53 3′-untranslated region mRNA or indirectly inhibit p53-modulator proteins, such as Mdm2 and Mdm4, thereby determining the cell fate of damaged/transformed cells [71, 95]. The wt p53 prevents cancer development by employing the miRNAs, and mutant p53 contributes to chemoresistance and metastasis by different miRNAs. miRNAs act as key effectors in the p53 network, determining the contribution to combat or enhance tumor development [71, 94, 95, 381]. Further studies are needed to precisely elucidate the mechanism whereby the oncogenic metabolism inhibits p53 to induce EMT.

The genetic inactivation of p53 is induced by increasing ROS and ROS-mediated oxidative DNA damage through the impairment of the electron transport chain. p53, which regulates mitochondrial metabolism and cellular redox, is genetically inactivated in high-grade glioma [376, 384–386]. Mitochondrial respiratory chain dysfunction is caused by inhibition of the complex I subunit or by reducing the mitochondrial DNA copy number, which leads to genetic loss of the p53 and induces the glycolytic switch. Therefore, alteration of mitochondrial metabolism leads to p53 genetic loss in an ROS-dependent manner, which contributes to malignant transformation [376].

Glutamine metabolism negatively regulates p53, which inhibits Snail stability by upregulating miRNA, thereby promoting EMT [40]. Glutamine metabolism plays a crucial role in EMT and the glycolytic switch. Glutamine metabolism inhibition via GLS1 shRNA, DON, or glutamine deprivation inhibits TGF-*β*/Wnt/Dlx-2/Snail-induced EMT and glycolytic switch. GLS1 knockdown also leads to inhibition of tumor growth and metastasis in vivo [40]. Glutamine metabolism inhibition and Dlx-2 shRNA lead to increased p53 expression, which induces the p53-dependent upregulation of Snail-targeting miRNAs (miR-23b, miR-29b, miR-30, miR-34, miR-125b, miR-148a, miR-153, miR-200, and miR-203) and then to decreased Snail mRNA levels [40]. These observations indicate that the Dlx-2/GLS1/glutamine metabolic axis induces Snail mRNA stability by repressing the p53-dependent regulation of Snail-targeting miRNAs, thereby promoting EMT [40].

As well as GLS, other enzymes active in oncogenic metabolism may also contribute to Snail-induced EMT by regulating the p53-dependent modulation of Snail-targeting miRNAs. In addition, abnormal TCA cycle enzymes, such as IDH, prevent p53 activities [335]. These results suggest that enzymes active in oncogenic metabolism and mitochondrial metabolism play important roles in mediating the inhibition of p53. Furthermore, oncogenic metabolism may inactivate p53 levels and function.

### 4.2. Regulation of EMT by Oncogenic Metabolism-Generated ROS/NADPH

Oncogenic metabolism can cause changes in the levels of ROS and NADPH, which may contribute to cancer metastasis and progression. Mutations in metabolic enzymes increase ROS production [335]. Indeed, mutations of IDH and SDH induce high levels of ROS that include superoxide anion, hydrogen peroxide, and hydrogen radicals. ROS stimulate oxidative stress in cells, which induces carcinogenesis [335, 352, 387, 388].

ROS play an important role in EMT. ROS and NADPH oxidase 2 are dramatically induced by TGF-*β* treatments. NADPH oxidase generates superoxide by transporting electrons from NADPH to oxygen. Conversely, TGF-*β*-induced EMT is inhibited by antioxidant treatment. In addition, the NADPH oxidase 2 complex and ROS production are activated by overexpression of neutrophil cytosolic factor 4. The latter encodes the p40phox polypeptide, which is the last NADPH oxidase subunit. In response to the expression of neutrophil cytosolic factor 4, expressions of several matrix metalloproteinases are increased. Snail, Slug, and vimentin are also increased and E-cadherin is decreased in response to neutrophil cytosolic factor 4 expression. Thus, ROS signaling is required for increased EMT [389].

Under conditions of metabolic stress, NADPH homeostasis is important in the survival of cancer cells. NADPH is mainly generated from the cytosolic oxidative pentose phosphate pathway and one-carbon metabolism. NADPH is necessary to scavenge ROS, which is generated with ATP during OXPHOS. Under oxidative and metabolic stress, Snail regulates glucose flux through the pentose phosphate pathway for cancer cell survival [390].

IDH mutation inhibits the production of NADPH and increases the consumption of NADPH [335, 391–393]. Mutation in FH induces accumulation of fumarate, which binds the antioxidant glutathione, and then produces the novel cancer metabolite succinated glutathione. Succinated glutathione can act as an alternative substrate to glutathione reductase, resulting in decreased NADPH levels. Interestingly, FH impaired or deficient cells display decreased levels of NADPH and high levels of ROS, which activate cancer metabolism [335, 348, 394].

Therefore, oncogenic metabolism may contribute to cancer metastasis and progression by the changes in the levels of ROS and NADPH.

## 5. Conclusions

Most (90%) of cancer deaths results from cancer that metastasized. Metastasis is the movement of cancer cells from the primary tumor to surrounding tissues and sometimes more distant organs [3, 16–23]. EMT is essential in the initiation of metastasis [5]. Cancer cells that undergo EMT exhibit enhanced metastatic ability, acquire CSC properties, and display metabolic alterations [3, 19–23]. These properties are closely interconnected and are important in determining outcomes of cancer treatment. Thus, targeting EMT, CSCs, and oncogenic metabolic pathways may prevent distant metastasis.

EMT is regulated by various transcription factors (Snail, ZEB1/2, Twist1/2, E12/E47, HIF-1*α*, and Dlx-2) and signaling factors (TGF-*β*, Wnt, Notch, Hedgehog, NF-*κ*B, ERK, and PI3K/Akt) [3, 17, 20–22, 24–27, 57–60]. Several EMT-inducing transcription factors are negatively regulated by p53 [80–82]. p53 is traditionally a tumor suppressor [71–78]. It is a master regulator of metastasis [79–87]. Indeed, the loss or mutation of p53 occurs frequently in human cancer and these events can affect the metastatic potential of tumor cells. The loss of p53 leads to the disruption of pathways that inhibit metastasis and p53 mutations can promote metastasis [80]. In addition, p53-dependent regulation of miRNAs and lncRNAs has been implicated in EMT and metastasis [71, 93–95, 103–105].

Numerous EMT-inducing transcription factors and EMT-related signaling pathways contribute to metastasis as well as the CSC phenotype [3, 17, 20–22, 24–27, 57–60, 108–114]. Mutant p53 gain-of-function is also associated with cell stemness [142].

Many regulatory molecules that are involved in EMT, including Snail, Dlx-2, HIF-1*α*, STAT3, TGF-*β*, Wnt, and Akt, have been implicated in the metabolic reprogramming of cancer cells. The induction of EMT leads to repression of mitochondrial metabolism and induction of the glycolytic switch [39, 40, 206, 240, 252–259]. In addition, metabolic reprogramming is involved in tumor development, particularly the acquisition of the invasive phenotype [395].

The Warburg effect induces an acidic environment, leading to ECM destruction and induction of metastasis [209, 286]. A glycolytic mechanism is also involved in the regulation of the angiogenic switch that effects metastatic growth [22, 287]. Several metabolic enzymes contribute to regulation of EMT, metastasis, and CSC phenotype [22, 40, 240, 267–285].

p53 directly regulates several metabolic enzymes that regulate the metabolic network. Under nutrient stress conditions, p53 inhibits anabolic metabolism to conserve cellular energy [361]. p53 is important in anoikis, which contributes to metastasis suppressor [80, 373]. Anoikis is a type of p53-dependent apoptosis that is caused by inadequate/inappropriate cell matrix interactions [374] and which is triggered by cell detachment from the ECM, leading to inhibition of adherent-independent cell growth and attachment to an inappropriate matrix, thereby repressing colonization of distant organs [375, 396]. Furthermore, enzymes in oncogenic metabolism, including mitochondrial metabolism, can play important roles in mediating the inhibition of p53, thereby promoting EMT and metastasis.

The collective findings presented in this review indicate that oncogenic metabolism can regulate metastasis and CSCs. Oncogenic metabolism may also be closely related with metastasis. Thus, oncogenic metabolism might be the major target for the prevention of metastasis. Understanding the molecular mechanisms between oncogenic metabolism and metastasis will reveal the role of the metabolism in tumor development and will be crucial for the development of therapeutic strategies.
